# Supplementary material for: Intranational synergies and trade-offs reveal common and differentiated priorities of sustainable development goals in China
Source: Nat Commun. 2024 Mar 13;15:2251. doi: 10.1038/s41467-024-46491-6 (PMC10937989; doi:10.1038/s41467-024-46491-6)
Supplement: Supplementary file 1 — Supplementary Information [file 41467_2024_46491_MOESM1_ESM.pdf]

Supplementary Information for

Intranational synergies and trade-offs reveal common and differentiated priorities of Sustainable Development Goals in China

Qiang Xing[1,2], Chaoyang Wu[3,4]*, Fang Chen[1,2,4]*, Jianguo Liu[5], Prajal Pradhan[6,7], Brett A Bryan[8], Thomas Schaubroeck[9], L. Roman Carrasco[10], Alemu Gonsamo[11], Yunkai Li[12], Xiuzhi Chen[12], Xiangzheng Deng[3,4], Andrea Albanese[13], Yingjie Li[5,14], Zhenci Xu[15]

Correspondence to: wucy@igsnrr.ac.cn, chenfang@radi.ac.cn

This PDF file includes:

Figs. 1-2
Table 1-3
Text
Reference

Other Supplementary Information for this manuscript includes the following:

Data for Synergies and trade-offs at the national and provincial levels
Explanations on the associations between indicators
Source data for the figures in main text
Source data for the figures in supplementary information
Original data at the national and provincial levels

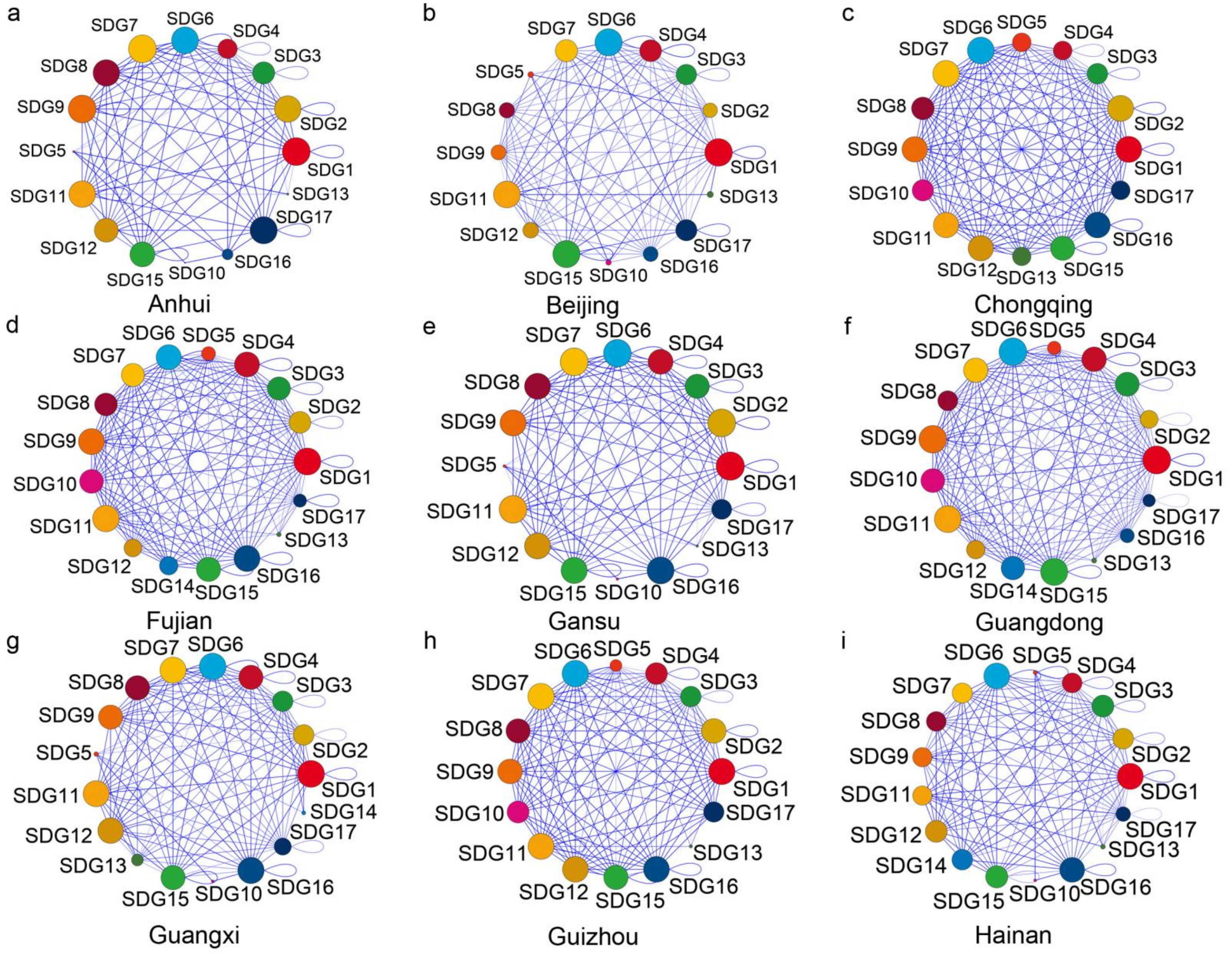
a
SDG7 SDG6 SDG4 SDG3 SDG8 SDG9 SDG2 SDG5 SDG1 SDG13 SDG11 SDG17 SDG12 SDG15 SDG10 SDG16
Anhui
b
SDG7 SDG6 SDG4 SDG3 SDG5 SDG2 SDG8 SDG9 SDG1 SDG11 SDG13 SDG12 SDG17 SDG15 SDG10 SDG16
Beijing
c
SDG6 SDG5 SDG4 SDG7 SDG3 SDG8 SDG2 SDG9 SDG1 SDG10 SDG17 SDG11 SDG16 SDG12 SDG13 SDG15
Chongqing
d
SDG6 SDG5 SDG4 SDG7 SDG3 SDG2 SDG8 SDG9 SDG1 SDG10 SDG17 SDG11 SDG13 SDG12 SDG14 SDG15 SDG16
Fujian
e
SDG7 SDG6 SDG4 SDG3 SDG8 SDG2 SDG9 SDG5 SDG1 SDG11 SDG17 SDG13 SDG12 SDG15 SDG10 SDG16
Gansu
f
SDG6 SDG5 SDG4 SDG7 SDG3 SDG8 SDG9 SDG2 SDG10 SDG1 SDG17 SDG11 SDG16 SDG13 SDG12 SDG14 SDG15
Guangdong
g
SDG7 SDG6 SDG4 SDG8 SDG3 SDG9 SDG2 SDG5 SDG1 SDG11 SDG14 SDG17 SDG12 SDG13 SDG15 SDG10 SDG16
Guangxi
h
SDG6 SDG5 SDG4 SDG3 SDG7 SDG8 SDG2 SDG9 SDG1 SDG10 SDG17 SDG11 SDG13 SDG12 SDG15 SDG16
Guizhou
i
SDG6 SDG5 SDG4 SDG3 SDG7 SDG8 SDG2 SDG9 SDG1 SDG11 SDG17 SDG12 SDG13 SDG14 SDG15 SDG10 SDG16
Hainan

Fig. 1. The synergy networks at the provincial level

The thickness of the edges in the synergy network indicates the weighted edge between SDG Pairs. The thicker the lines are, the stronger the strengths are. The size of the circle suggests its importance as a central hub in the network. The larger the circle is, the more important the node as a central hub is. In the synergy network the edge lines is shown in blue. The background color of each node is consistent with the SDG icon of the United Nations (UN). The 31 synergy networks at sub-national level in alphabetical order. (a) is for Anhui (0.2-1), (b) is for Beijing (0.18-1), (c) is for Chongqing (0.4-1), (d) is for Fujian (0.11-0.99), (e) is for Gansu (0.1-1), (f) is for Guangdong (0.14-0.99), (g) is for Guangxi (0.22-1), (h) is for Guizhou (0.15-1), (i) is for Hainan (0.14-1), continued,

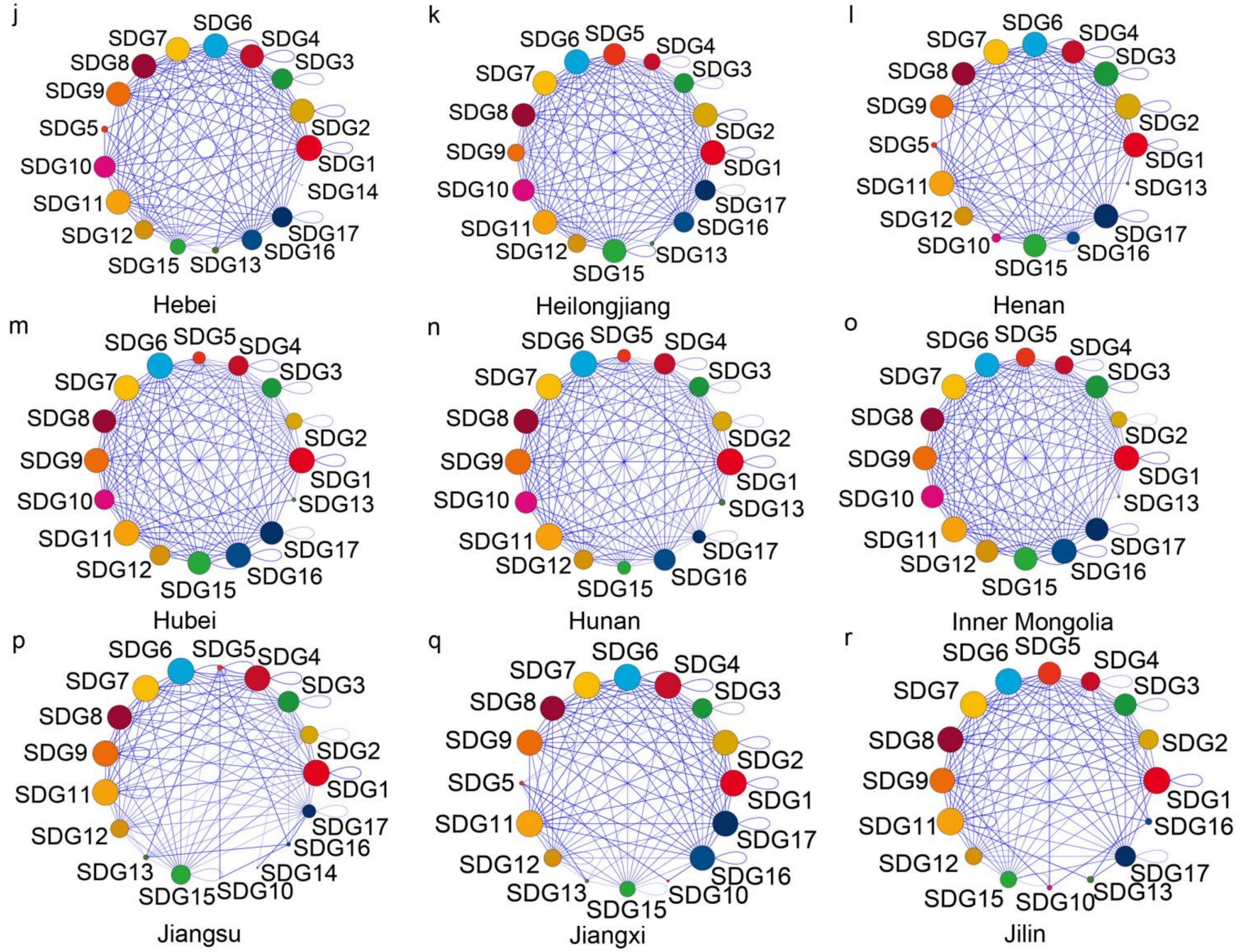

(j) is for Hebei (0.3-1), (k) is for Heilongjiang (0.3-1), (l) is for Henan (0.29-1), (m) is for Hubei (0.24-1), (n) is for Hunan (0.14-1), (o) is for Inner Mongolia (0.13-1), (p) is for Jiangsu (0.14-1), (q) is for Jiangxi (0.23-1), (r) is for Jilin (0.3-1), continued,

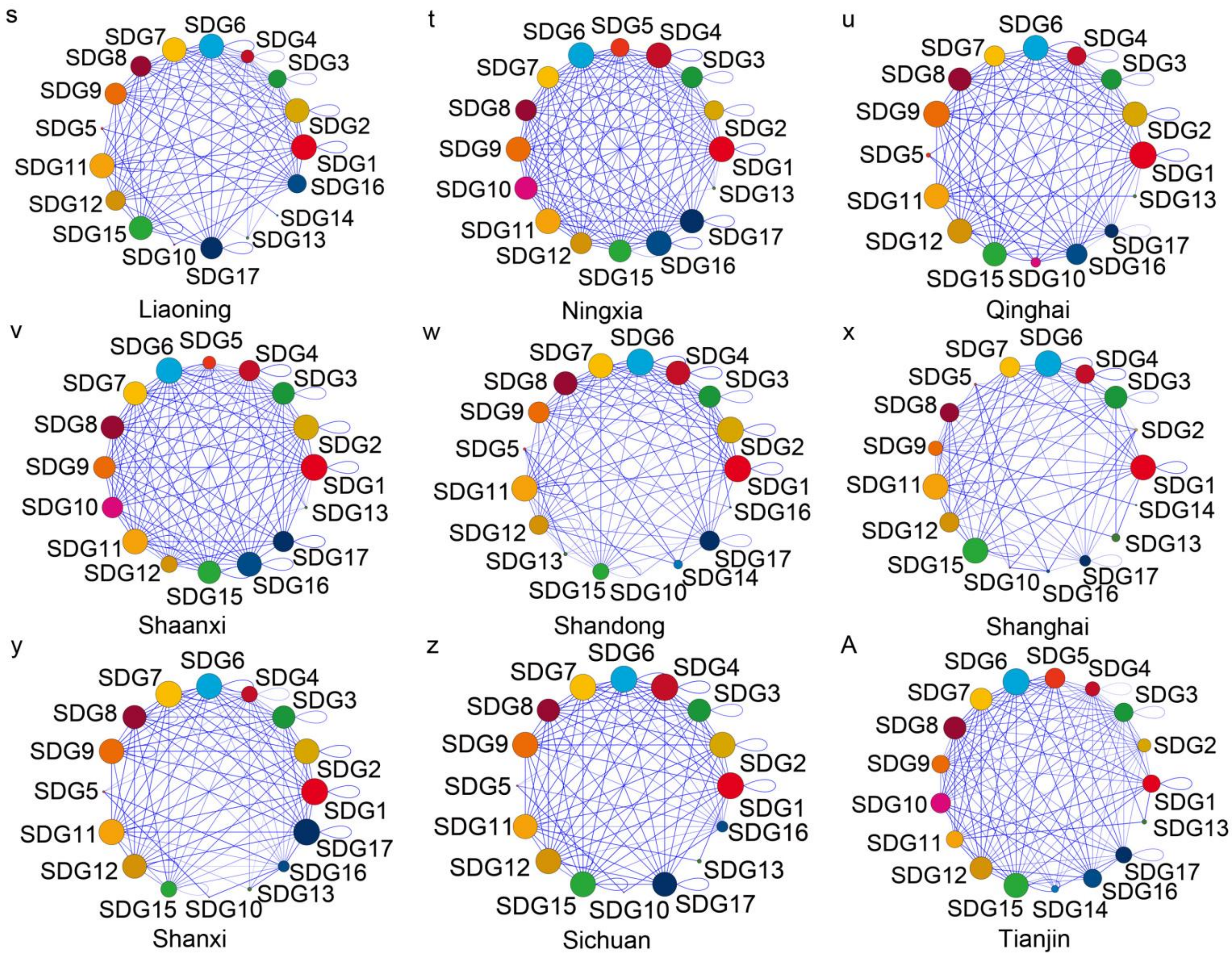

(s) is for Liaoning (0.15-1), (t) is for Ningxia (0.14-1), (u) is for Qinghai (0.29-1), (v) is for Shaanxi (0.1-1), (w) is for Shandong (0.12-1), (x) is for Shanghai (0.17-1), (y) is for Shanxi (0.15-1), (z) is for Sichuan (0.11-1), (A) is for Tianjin (0.23-1), continued,

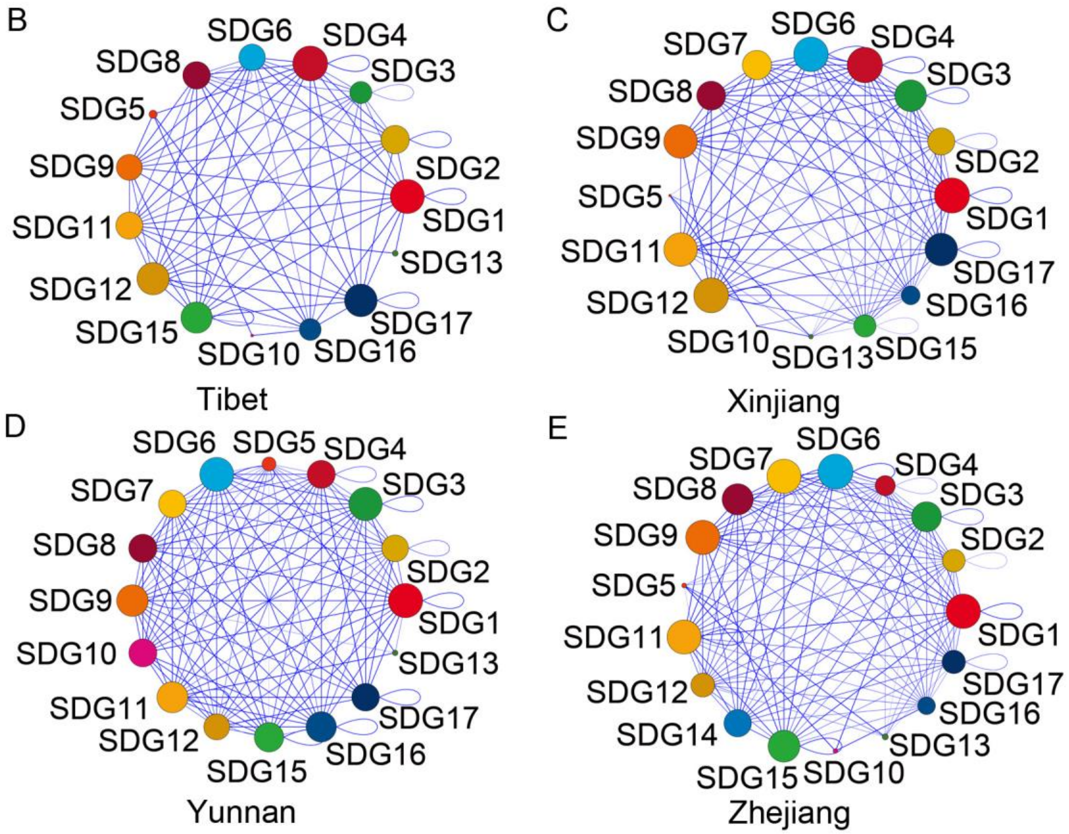

(B) is for Tibet (0.46-1), (C) is for Xinjiang (0.13-1), (D) is for Yunnan (0.19-1) and (E) is for Zhejiang (0.13-1).

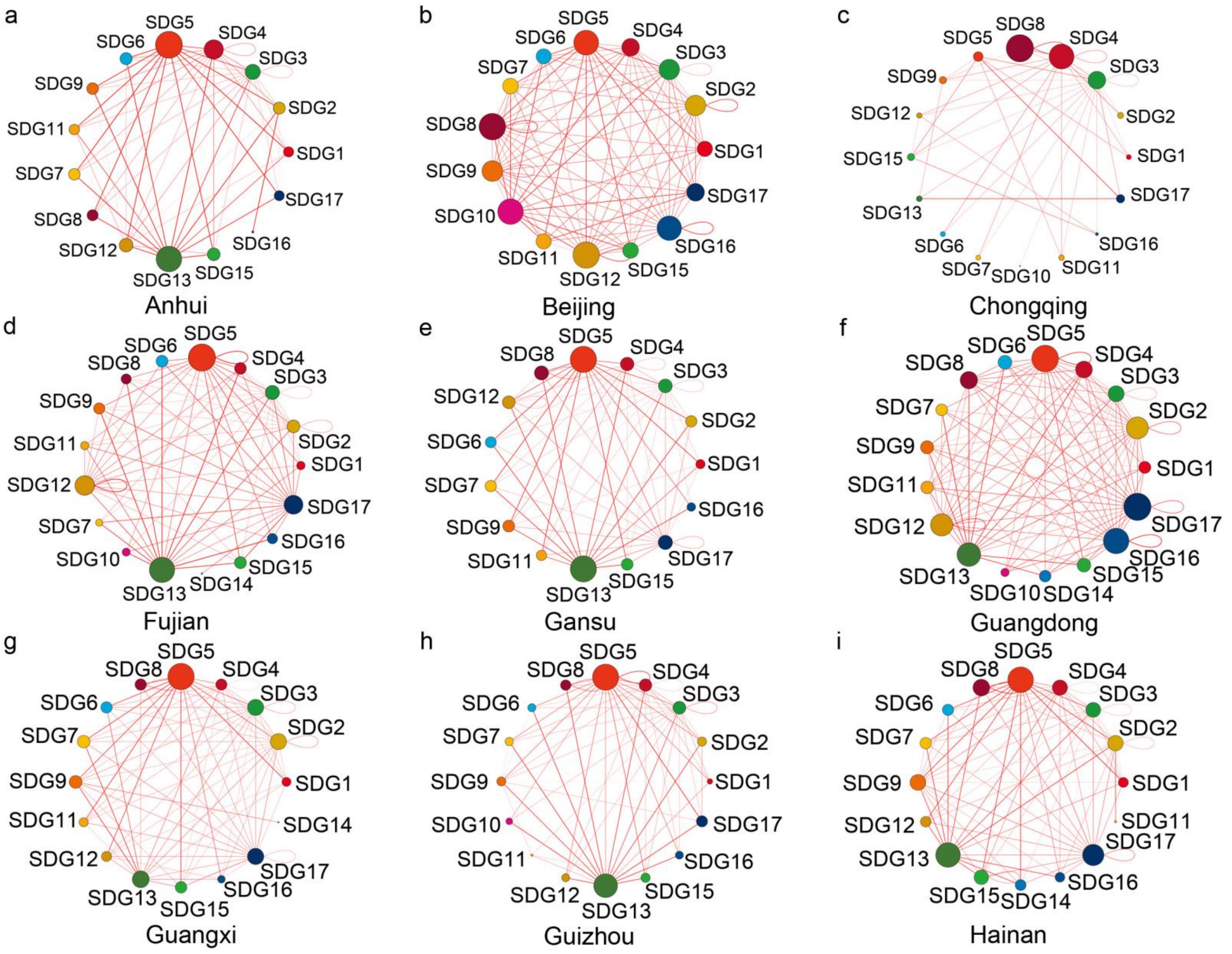
a
SDG5
SDG4
SDG3
SDG6
SDG9
SDG2
SDG11
SDG1
SDG7
SDG17
SDG8
SDG16
SDG12
SDG13
SDG15
Anhui
b
SDG5
SDG4
SDG6
SDG3
SDG7
SDG2
SDG8
SDG1
SDG9
SDG17
SDG10
SDG16
SDG11
SDG12
SDG15
Beijing
c
SDG8
SDG5
SDG4
SDG3
SDG9
SDG12
SDG2
SDG1
SDG15
SDG17
SDG13
SDG16
SDG6
SDG7
SDG10
SDG11
Chongqing
d
SDG5
SDG6
SDG4
SDG8
SDG3
SDG9
SDG2
SDG11
SDG1
SDG12
SDG17
SDG7
SDG16
SDG10
SDG15
SDG13
SDG14
Fujian
e
SDG5
SDG4
SDG8
SDG3
SDG12
SDG2
SDG6
SDG1
SDG7
SDG16
SDG9
SDG17
SDG11
SDG15
SDG13
Gansu
f
SDG6
SDG5
SDG4
SDG8
SDG3
SDG7
SDG2
SDG9
SDG1
SDG11
SDG17
SDG12
SDG16
SDG13
SDG15
SDG10
SDG14
Guangdong
g
SDG5
SDG8
SDG4
SDG3
SDG6
SDG2
SDG7
SDG9
SDG1
SDG11
SDG14
SDG12
SDG17
SDG16
SDG13
SDG15
Guangxi
h
SDG5
SDG8
SDG4
SDG3
SDG6
SDG7
SDG2
SDG9
SDG1
SDG10
SDG17
SDG11
SDG16
SDG15
SDG12
SDG13
Guizhou
i
SDG5
SDG8
SDG4
SDG3
SDG6
SDG2
SDG7
SDG9
SDG1
SDG12
SDG11
SDG17
SDG13
SDG16
SDG15
SDG14
Hainan

Fig. 2. The trade-off networks at the provincial level

The thickness of the edge lines in the trade-off network indicates the weighted edge between SDG Pairs. The thicker the lines are, the stronger the strengths are. The size of the circle suggests its importance as a central hub in the network. The larger the circle is, the more important the node as a central hub is. In the trade-off network the edge lines is shown red. The background color of each node is consistent with the SDG icon of the United Nations (UN). The 31 trade-off networks at sub-national level in alphabetical order. (a) is for Anhui (0.05-1), (b) is for Beijing (0.18-1), (c) is for Chongqing (0.11-0.9), (d) is for Fujian (0.09-0.97), (e) is for Gansu (0.06-0.97), (f) is for Guangdong (0.08-1), (g) is for Guangxi (0.1-0.95), (h) is for Guizhou (0.07-0.98), (i) is for Hainan (0.07-0.99), continued,

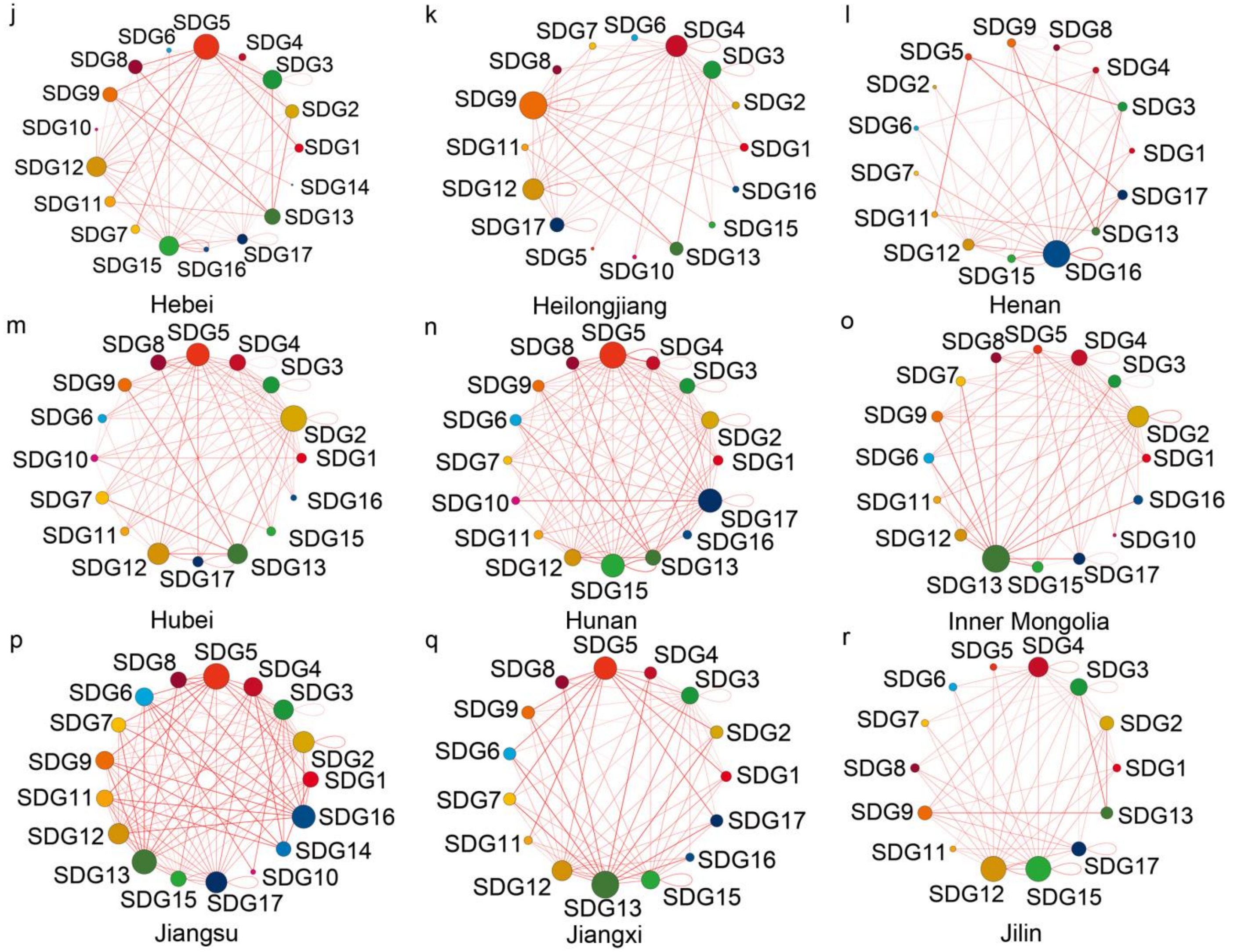

(j) is for Hebei (0.08-0.91), (k) is for Heilongjiang (0.12-0.87), (l) is for Henan (0.04-0.93), (m) is for Hubei (0.09-0.86), (n) is for Hunan (0.12-0.94), (o) is for Inner Mongolia (0.04-0.97), (p) is for Jiangsu (0.08-1), (q) is for Jiangxi (0.06-1), (r) is for Jilin (0.11-0.93), continued,

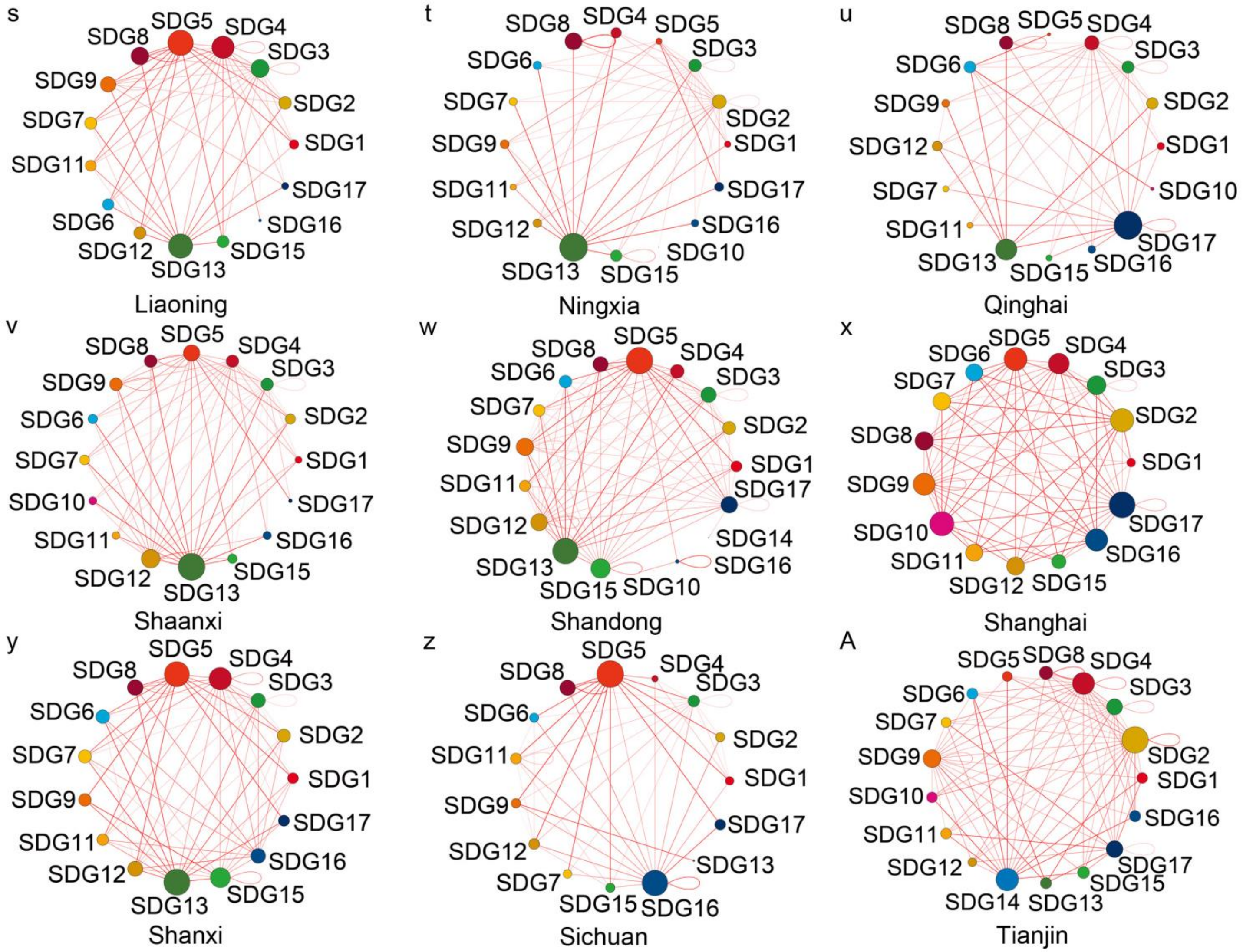

(s) is for Liaoning (0.13-0.92), (t) is for Ningxia (0.11-0.98), (u) is for Qinghai (0.11-1), (v) is for Shaanxi (0.09-0.97), (w) is for Shandong (0.09-1), (x) is for Shanghai (0.22-0.98), (y) is for Shanxi (0.09-1), (z) is for Sichuan (0.09-1), (A) is for Tianjin (0.2-0.94), continued,

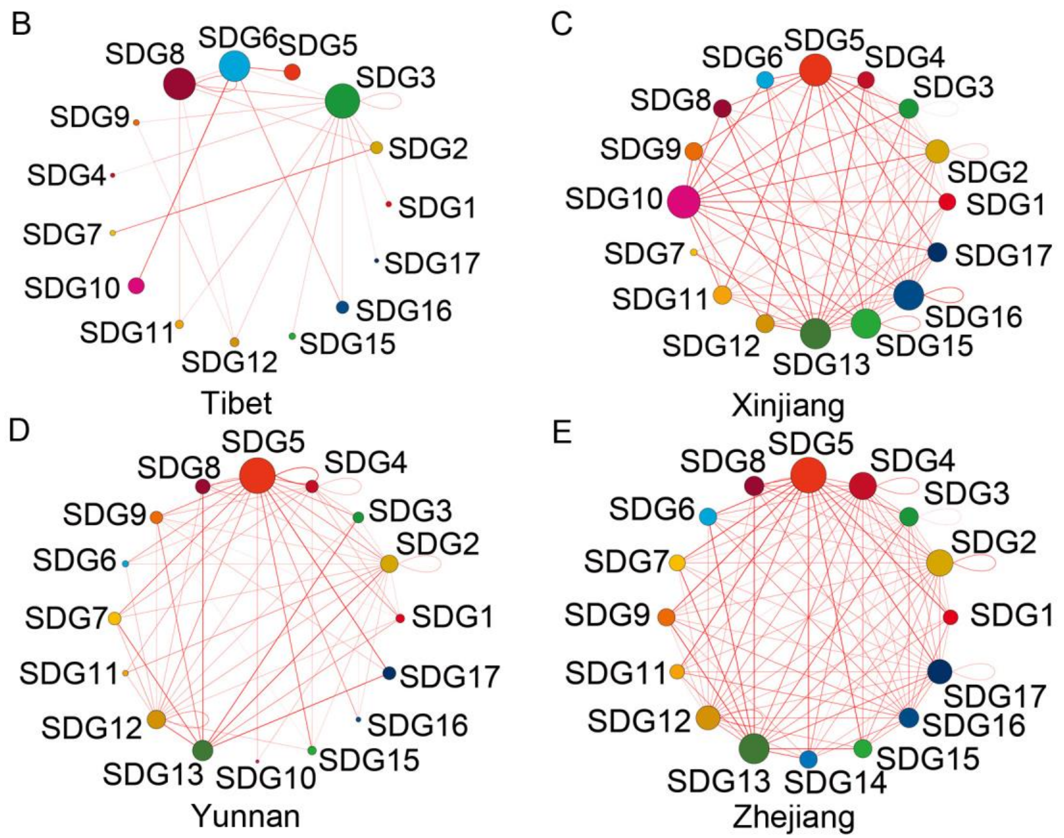

(B) is for Tibet (0.14-1), (C) is for Xinjiang (0.07-0.99), (D) is for Yunnan (0.03-0.96) and (E) is for Zhejiang (0.1-1).

Table 1.

The averaged correlation coefficient (R) and Number of indicator pairs (N) for synergy, weak synergy, trade-off and weak trade-off groups at the provincial level.

Provinces	Correlation coefficient (R)				Number of indicator pairs (N)			
	Synergy	Weak Synergy	Trade-off	Weak Trade-off	Synergy	Weak Synergy	Trade-off	Weak Trade-off
Anhui	0.94	0.50	-0.93	-0.43	872	2235	238	1206
Beijing	0.94	0.40	-0.93	-0.38	251	2190	184	1886
Chongqing	0.94	0.53	-0.91	-0.46	773	2144	126	1589
Fujian	0.94	0.46	-0.94	-0.40	757	2264	235	1581
Gansu	0.94	0.51	-0.93	-0.46	763	2197	171	1514
Guangdong	0.94	0.49	-0.94	-0.46	660	2054	305	1819
Guangxi	0.93	0.47	-0.91	-0.41	721	2270	242	1608
Guizhou	0.94	0.48	-0.93	-0.42	748	2295	174	1412
Hainan	0.93	0.47	-0.91	-0.42	438	2275	207	1822
Hebei	0.96	0.48	-0.92	-0.40	794	2370	167	1607
Heilongjiang	0.93	0.48	-0.91	-0.43	641	2167	147	1685
Henan	0.94	0.49	-0.90	-0.43	677	2513	96	1360
Hubei	0.94	0.49	-0.91	-0.39	710	2050	216	1666
Hunan	0.95	0.50	-0.92	-0.46	820	2216	380	1136
Inner Mongolia	0.93	0.49	-0.93	-0.42	768	2135	185	1560
Jiangsu	0.94	0.55	-0.94	-0.48	524	2259	291	1760
Jiangxi	0.95	0.52	-0.94	-0.45	674	2026	208	1644
Jilin	0.93	0.43	-0.93	-0.42	476	2339	138	1689
Liaoning	0.93	0.41	-0.92	-0.39	489	2562	202	1680
Ningxia	0.94	0.50	-0.92	-0.42	567	2131	108	1743
Qinghai	0.95	0.50	-0.90	-0.46	503	2442	154	1448
Shaanxi	0.94	0.52	-0.93	-0.43	683	2229	179	1556
Shandong	0.94	0.51	-0.92	-0.47	654	2217	247	1725
Shanghai	0.93	0.44	-0.91	-0.42	244	2122	208	1957
Shanxi	0.94	0.46	-0.92	-0.40	586	2070	213	1778
Sichuan	0.94	0.52	-0.94	-0.44	721	2191	133	1604
Tianjin	0.92	0.43	-0.91	-0.39	279	2283	174	1986
Tibet	0.94	0.45	-0.93	-0.38	324	2127	62	1541
Xinjiang	0.93	0.46	-0.93	-0.39	460	2237	168	1671
Yunnan	0.95	0.49	-0.92	-0.40	789	2262	180	1417
Zhejiang	0.94	0.50	-0.93	-0.48	680	2309	329	1397

Table 2.

Information about the indictors, targets and goals in the study. The name of the indicators is listed in the third column. For coding of the indicators, if the name of the indicator cannot be found in the official document of United Nations, the code of indicator is selected based on its closeness to the definition of the target and goal with the alphabet "L" added. The positive and negative sign in the fourth column refers to judging whether the higher the value of each indicator is, the better it is to promote sustainable development or the lower the value is, the better it is to promote sustainable development. The data source of every indicator is listed in the fifth column. The indicator source is listed in the sixth column. The maximum time range of the national and provincial data is listed in the seventh column. For detailed time range of the national and 31 provinces, please refer to the data file in excel format.

SDG	Target	Indicators	Positive or Negative	Data sources	Indicator sources	Year
SDG1 No Poverty	1.2 By 2030, reduce at least by half the proportion of men, women and children of all ages living in poverty in all its dimensions according to national definitions	1.2.1 Rural poverty incidence rate	-1	Poverty Monitoring Report of Rural China (2020)[1]	Zhang et al., 2022[21]	2010-2019
	1.3 Implement nationally appropriate social protection systems and measures for all, including floors, and by 2030 achieve substantial coverage of the poor and the vulnerable	1.3.1 Proportion of population covered by insurance (endowment, unemployment, and medicare)	1	China Statistical Yearbook, Yearbook of China's Insurance [2,3]	Xu et al., 2020[22]	2000-2020
	1.4 By 2030, ensure that all men and women, in particular the poor and the vulnerable, have equal rights to economic resources, as well as access to basic services, ownership and control over land and other forms of property, inheritance, natural resources, appropriate new technology and financial	1.4.1 Proportion of population with access to basic services such as education and insurance	1	China Statistical Yearbook, Yearbook of China's Insurance [2,3]	Xu et al., 2020[22]	2000-2020

	services, including microfinance					
	1.5 By 2030, build the resilience of the poor and those in vulnerable situations and reduce their exposure and vulnerability to climate-related extreme events and other economic, social and environmental shocks and disasters	1.5.1 Number of deaths, missing persons and directly affected persons attributed to disasters per persons attributed to disasters per 100,000 population	-1	China Statistical Yearbook on Environment, China Statistical Yearbook[2,4]	United Nations Statistics Division, 2023[23]	2004-2020
		1.5.2 Direct economic loss attributed to disasters in relation to global gross domestic product (GDP)	-1	China Statistical Yearbook on Environment, China Statistical Yearbook[2,4]	United Nations Statistics Division, 2023[23]	2004-2020
		1.5.4 Proportion of local governments that adopt and implement local disaster risk reduction strategies in line with national disaster risk reduction strategies	1	China Statistical Yearbook on Environment[5]	United Nations Statistics Division, 2023[23]	2001-2020
SDG2 Zero Hunger	2.1 By 2030, end hunger and ensure access by all people, in particular the poor and people in vulnerable situations, including infants, to safe,	2.1.2 Cereal yield (t/ha)	1	China Statistical Yearbook[2]	Sustainable Developme nt Report 2022[24]	2000-2020

	nutritious and sufficient food all year round					
	2.2 By 2030, end all forms of malnutrition, including achieving, by 2025, the internationally agreed targets on stunting and wasting in children under 5 years of age, and address the nutritional needs of adolescent girls, pregnant and lactating women and older persons	2.2.1 Percentage children born with low birth weight	-1	China Health Statistical Yearbook [6]	Sustainable Development Solutions Network, 2015[25]	2002-2020
		2.2.2 Prevalence of malnutrition among children under 5 years of age	-1	China Health Statistical Yearbook [6]	United Nations Statistics Division, 2023[23]	2002-2020
	2.3 By 2030, double the agricultural productivity and incomes of small-scale food producers, in particular women, indigenous peoples, family farmers, pastoralists and fishers, including through secure and equal access to land, other productive resources and inputs, knowledge, financial services, markets and opportunities for value addition and non-farm employment	2.3.1 Volume of production (farming/pastoral/forestry) per capita (rural population)	1	China Rural Statistical Yearbook, China Population & employment Statistics Yearbook [7,8]	Xu et al., 2020[22]	2000-2020

	2.4 By 2030, ensure sustainable food production systems and implement resilient agricultural practices that increase productivity and production, that help maintain ecosystems, that strengthen capacity for adaptation to climate change, extreme weather, drought, flooding and other disasters and that progressively improve land and soil quality	2.4.1 Proportion of agricultural area under productive and sustainable agriculture	1	China Statistical Yearbook[2]	Author constructed	2000-2020
	2.a Increase investment, including through enhanced international cooperation, in rural infrastructure, agricultural research and extension services, technology development and plant and livestock gene banks in order to enhance agricultural productive capacity in developing countries, in particular least developed countries	2.a.L Number of agricultural extension workers per 1,000 farmers	1	China Statistical Yearbook on Science and Technology, China Population & employment Statistics Yearbook[7,9]	Sustainable Development Solutions Network, 2015[25]	2000-2017
SDG3 Good Health and Well-being	3.1 By 2030, reduce the global maternal mortality ratio to less than 70 per 100,000 live births	3.1.1 Maternal mortality ratio	-1	China Health Statistical Yearbook[6]	United Nations Statistics Division, 2023[23]	2000-2020
		3.1.2 Proportion of births attended by	1	China Health and Family Planning	United Nations	2002-2020

		skilled health personnel		Statistical Yearbook [10]	Statistics Division, 2023[23]	
	3.2 By 2030, end preventable deaths of newborns and children under 5 years of age, with all countries aiming to reduce neonatal mortality to at least as low as 12 per 1,000 live births and under-5 mortality to at least as low as 25 per 1,000 live births	3.2.1 Under-five child with low birth weight (%)	-1	China Health Statistical Yearbook [6]	Author constructed	2002-2020
		3.2.2 Neonatal mortality rate (per 1,000 live births)	-1	China Health Statistical Yearbook [6]	United Nations Statistics Division, 2023[23]	2002-2020
	3.3 By 2030, end the epidemics of AIDS, tuberculosis, malaria and neglected tropical diseases and combat hepatitis, water-borne diseases and other communicable diseases	3.3.1 Number of new HIV infections per 100,000 uninfected population	-1	China Health Statistical Yearbook [6]	United Nations Statistics Division, 2023[23]	2002-2020
		3.3.2 Tuberculosis incidence per 100,000 population	-1	China Health Statistical Yearbook [6]	United Nations Statistics Division, 2023[23]	2002-2020
		3.3.3 Malaria incidence per 100,000 population	-1	China Health Statistical Yearbook [6]	United Nations Statistics Division, 2023[23]	2002-2020
		3.3.4 Hepatitis B incidence per 100,000 population	-1	China Health Statistical Yearbook [6]	United Nations Statistics Division,	2002-2020

					2023[23]	
	3.4 By 2030, reduce by one third premature mortality from non-communicable diseases through prevention and treatment and promote mental health and well-being	3.4.L Annual consumption of vegetables by rural population (kg/year/person)	1	China Statistical Yearbook[2]	Xu et al., 2020[22]	2000-2012, 2015-2020
	3.6 By 2020, halve the number of global deaths and injuries from road traffic accidents	3.6.1 Death rate due to road traffic injuries (per 100,000 people)	-1	China Statistical Yearbook[2]	United Nations Statistics Division, 2023[23]	2000-2020
	3.8 Achieve universal health coverage, including financial risk protection, access to quality essential health-care services and access to safe, effective, quality and affordable essential medicines and vaccines for all	3.8.1 Percentage of population without effective financial protection for health care	-1	China Statistical Yearbook[2]	Sustainable Developme nt Solutions Network, 2015[25]	2002-2020
		3.8.2 Expenditures on health as a share of total household income (urban population)	1	China Statistical Yearbook[2]	Xu et al., 2020[22]	2000-2020
	3.9 By 2030, substantially reduce the number of deaths and illnesses from hazardous chemicals and air, water and soil pollution and contamination	3.9.1 Keshan disease incidence per 100,000 population	-1	China Health Statistical Yearbook[6]	Zhang et al., 2022[21]	2002-2020
	3.b Support the research and development of vaccines and medicines for the communicable and non-communicable diseases that primarily affect	3.b.2 Total net official development assistance to medical research and basic health sectors as a proportion of government	1	Finance Yearbook of China[11]	United Nations Statistics Division, 2023[23]	2000-2020

	developing countries, provide access to affordable essential medicines and vaccines, in accordance with the Doha Declaration on the TRIPS Agreement and Public Health, which affirms the right of developing countries to use to the full the provisions in the Agreement on Trade-Related Aspects of Intellectual Property Rights regarding flexibilities to protect public health, and, in particular, provide access to medicines for all	expenditure budget				
		3.b.3 Percentage of health facilities meeting service specific readiness requirements (women and children care agencies)	1	China Health Statistical Yearbook[6]	Xu et al., 2020[22]	2002-2020
	3.c Substantially increase health financing and the recruitment, development, training and retention of the health workforce in developing countries, especially in least developed countries and small island developing States	3.c.1 Health worker density	1	China Health Statistical Yearbook[6]	United Nations Statistics Division, 2023[23]	2002-2020
SDG4 Quality Education	4.1 By 2030, ensure that all girls and boys complete free, equitable and quality primary and secondary education leading to relevant and effective learning outcomes	4.1.1 Net primary enrolment rate (%)	1	Educational Statistics Yearbook of China[12]	Sustainable Development Report 2022[24]	2002, 2010-2020
		4.1.2 Completion rate (primary education, lower secondary education, upper	-1	China Statistical Yearbook[2]	Author constructed	2000-2020

		secondary education) (%)				
	4.3 By 2030, ensure equal access for all women and men to affordable and quality technical, vocational and tertiary education, including university	4.3.1 Participation rate of youth and adults in formal education and training in the previous 12 months	1	Educational Statistics Yearbook of China[12]<br>China Statistical Yearbook[2]	Xu et al., 2020[22]	2004-2020
	4.4 By 2030, substantially increase the number of youth and adults who have relevant skills, including technical and vocational skills, for employment, decent jobs and entrepreneurship	4.4.1 Proportion of youth and adults with internet	1	China Statistical Yearbook[2]	Xu et al., 2020[22]	2011-2020
	4.5 By 2030, eliminate gender disparities in education and ensure equal access to all levels of education and vocational training for the vulnerable, including persons with disabilities, indigenous peoples and children in vulnerable situations	4.5.1 Ratio of female to male illiteracy rate	-1	China Population & Employment Statistic Yearbook[7]	Author constructed	2001-2020
	4.6 By 2030, ensure that all youth and a substantial proportion of adults, both men and women, achieve literacy and numeracy	4.6.1 Proportion of population achieving at least a fixed level of proficiency in functional literacy skills	1	Educational Statistics Yearbook of China[12]<br>China Statistical Yearbook[2]	United Nations Statistics Division, 2023[23]	2000-2020

	4.a Build and upgrade education facilities that are child, disability and gender sensitive and provide safe, non-violent, inclusive and effective learning environments for all	4.a.L Number of computers per school: (a) primary; (b) lower secondary; and (c) upper secondary education	1	Educational Statistics Yearbook of China[12]	Xu et al., 2020[22]	2002-2019
	4.b By 2020, substantially expand globally the number of scholarships available to developing countries, in particular least developed countries, small island developing States and African countries, for enrolment in higher education, including vocational training and information and communications technology, technical, engineering and scientific programmes, in developed countries and other developing countries	4.b.L Government expenditure on education (% total government expenditure)	1	Finance Yearbook of China[11]	Xu et al., 2020[22]	2000-2020
	4.c By 2030, substantially increase the supply of qualified teachers, including through international cooperation for teacher training in developing countries, especially least developed countries and small island developing States	4.c.1 Proportion of teachers in: (a) preprimary; (b) primary; (c) lower secondary; and (d) upper secondary education who have got a bachelor degree or above	1	Educational Statistics Yearbook of China[12]	Xu et al., 2020[22]	2002-2019

SDG5 Gender Equality	5.3 Eliminate all harmful practices, such as child, early and forced marriage and female genital mutilation	5.3.2 Ratio of female to male sterilization rate	-1	China Population & Employment Statistic Yearbook[7]	Xu et al., 2020[22]	2000-2017
	5.5 Ensure women's full and effective participation and equal opportunities for leadership at all levels of decision-making in political, economic and public life	5.5.1 Proportion of seats held by women in parliaments (national and provincial people's congress in China)	1	National People's Congress of the People's Republic of China[13]	United Nations Statistics Division, 2023[23]	2003-2017
		5.5.2L Ratio of male to female employment rate	-1	China Population & Employment Statistic Yearbook[7]	Xu et al., 2020[22]	2001-2020
	5.a Undertake reforms to give women equal rights to economic resources, as well as access to ownership and control over land and other forms of property, financial services, inheritance and natural resources, in accordance with national laws	5.a.1 Share of women among owners or rights-bearers of agricultural land	1	China Population & Employment Statistic Yearbook[7]	United Nations Statistics Division, 2023[23]	2003-2020
	5.b Enhance the use of enabling technology, in particular information and communications technology, to promote the empowerment of women	5.b.L Ratio of female to male illiteracy rate	-1	China Population & Employment Statistic Yearbook[7]	Xu et al., 2020[22]	2001-2020
SDG6 Clean Water and Sanitation	6.1 By 2030, achieve universal and equitable access to safe and affordable drinking water for all	6.1.1 The proportion of the population benefiting from the treated water - Endemic fluorosis (water type) per 100,000 population	1	China Health Statistical Yearbook[6]	Zhang et al., 2022[21]	2002-2020

	6.2 By 2030, achieve access to adequate and equitable sanitation and hygiene for all and end open defecation, paying special attention to the needs of women and girls and those in vulnerable situations	6.2.1 Popularity rate of sanitary toilet in rural area	1	China Rural Statistical Yearbook [8]	Xu et al., 2020[22]	2002-2017
	6.3 By 2030, improve water quality by reducing pollution, eliminating dumping and minimizing release of hazardous chemicals and materials, halving the proportion of untreated wastewater and substantially increasing recycling and safe reuse globally	6.3.1 Sewage treatment rate in cities (%)	1	China Urban Construction Statistical Yearbook [14]	Zhang et al., 2022[21]	2002-2020
	6.4 By 2030, substantially increase water-use efficiency across all sectors and ensure sustainable withdrawals and supply of freshwater to address water scarcity and substantially reduce the number of people suffering from water scarcity	6.4.1 Water-use efficiency (Water consumption per GDP)	-1	China Statistical Yearbook[2]	Xu et al., 2020[22]	2002-2020
		6.4.2 Level of water stress: freshwater withdrawal as a proportion of available freshwater resources	-1	China Statistical Yearbook[2]	United Nations Statistics Division, 2023[23]	2002-2020
	6.6 By 2020, protect and restore water-related ecosystems, including mountains, forests, wetlands, rivers, aquifers and lakes	6.6.1 Wetland area as a proportion of total land area	1	China Statistical Yearbook on Environment, China Statistical Yearbook [2,4]	Author constructed	2001-2019

SDG7 Affordable and Clean Energy	7.1 By 2030, ensure universal access to affordable, reliable and modern energy services	7.1.2 Proportion of population with access to gas in cities (%)	1	China Statistical Yearbook[2]	Zhang et al., 2022[21]	2001-2020
	7.2 By 2030, increase substantially the share of renewable energy in the global energy mix	7.2.1 Proportion of clean energy power generation to total power generation (%)	1	China Energy Statistical Yearbook [15]	Zhang et al., 2022[21]	2000-2020
	7.3 By 2030, double the global rate of improvement in energy efficiency	7.3.1 Energy intensity measured in terms of primary energy and GDP	-1	China Energy Statistical Yearbook, China Statistical Yearbook[2,15]	United Nations Statistics Division, 2023[23]	2000-2019
SDG8 Decent Work and Economic Growth	8.1 Sustain per capita economic growth in accordance with national circumstances and, in particular, at least 7 per cent gross domestic product growth per annum in the least developed countries	8.1.1 Annual growth rate of real GDP per capita		China Statistical Yearbook[2]	Zhang et al., 2022[21]	2001-2020
	8.2 Achieve higher levels of economic productivity through diversification, technological upgrading and innovation, including through a focus on high-value added and labour-intensive sectors	8.2.1 Annual growth rate of real GDP per employed person	1	China Statistical Yearbook[2]	United Nations Statistics Division, 2023[23]	2001-2020

	8.4 Improve progressively, through 2030, global resource efficiency in consumption and production and endeavour to decouple economic growth from environmental degradation, in accordance with the 10-Year Framework of Programmes on Sustainable Consumption and Production, with developed countries taking the lead	8.4.2 Resource (water, energy) consumption per capita	-1	China Energy Statistical Yearbook, China Statistical Yearbook[2,15]	Xu et al., 2020[22]	2000-2020
	8.8 Protect labour rights and promote safe and secure working environments for all workers, including migrant workers, in particular women migrants, and those in precarious employment	8.8.1 Fatal and non-fatal occupational injuries treatment per 10,000 employed persons	1	China Statistical Yearbook[2]	Xu et al., 2020[22]	2000-2020
	8.9 By 2030, devise and implement policies to promote sustainable tourism that creates jobs and promotes local culture and products	8.9.1 Tourism direct GDP as a proportion of total GDP	1	Yearbook of China Tourism Statistics, China Statistical Yearbook [2,16]	United Nations Statistics Division, 2023[23]	2000-2020
SDG9 Industry, Innovation and Infrastructure	9.1 Develop quality, reliable, sustainable and resilient infrastructure, including regional and transborder infrastructure, to support economic development and human well-being, with a focus on affordable and equitable access for all	9.1.2 Passenger and freight volumes, by mode of transport (railway, highway, water transport)	1	Yearbook of China Transportation & Communications, China Statistical Yearbook[2,17]	United Nations Statistics Division, 2023[23]	2000-2020

	9.2 Promote inclusive and sustainable industrialization and, by 2030, significantly raise industry's share of employment and gross domestic product, in line with national circumstances, and double its share in least developed countries	9.2.1 Manufacturing value added as a proportion of GDP	1	China Statistical Yearbook[2]	United Nations Statistics Division, 2023[23]	2000-2020
		9.2.2 Manufacturing employment as a proportion of total employment	1	China Population & Employment Statistic Yearbook[7]	United Nations Statistics Division, 2023[23]	2000-2020
	9.3 Increase the access of small-scale industrial and other enterprises, in particular in developing countries, to financial services, including affordable credit, and their integration into value chains and markets	9.3.1 Proportion of small-scale industries in total industry value added	1	China Industry Statistical Yearbook [18]	United Nations Statistics Division, 2023[23]	2001-2003, 2005-2016, 2019-2020
	9.4 By 2030, upgrade infrastructure and retrofit industries to make them sustainable, with increased resource-use efficiency and greater adoption of clean and environmentally sound technologies and industrial processes, with all countries taking action in accordance with their respective capabilities	9.4.1 CO_2 emission per unit of value	-1	China Statistical Yearbook, Shan. et al., 2018, 2020, Guan et al. 2021 [2,19-21]	Xu et al., 2020[22]	2000-2019
	9.5 Enhance scientific research, upgrade the technological capabilities of	9.5.1 Research and development expenditure as a	1	China Statistical Yearbook on Science and	United Nations Statistics	2000-2020

	industrial sectors in all countries, in particular developing countries, including, by 2030, encouraging innovation and substantially increasing the number of research and development workers per 1 million people and public and private research and development spending	proportion of GDP		Technology, China Statistical Yearbook [2,9]	Division, 2023[23]	
		9.5.2 Researchers (in full-time equivalent) as a proportion of total population	1	China Statistical Yearbook on Science and Technology, China Statistical Yearbook [2,9]	Xu et al., 2020[22]	2000-2020
	9.c Significantly increase access to information and communications technology and strive to provide universal and affordable access to the Internet in least developed countries by 2020	9.c.1 Proportion of population covered by a mobile network	1	China Statistical Yearbook[2]	United Nations Statistics Division, 2023[23]	2014-2020
SDG10 Reduced Inequalities	10.2 By 2030, empower and promote the social, economic and political inclusion of all, irrespective of age, sex, disability, race, ethnicity, origin, religion or economic or other status	10.2.L Share of women among owners or rights-bearers of agricultural land	1	China Population & Employment Statistic Yearbook[7]	Xu et al., 2020[22]	2003-2020
	10.3 Ensure equal opportunity and reduce inequalities of outcome, including by eliminating discriminatory laws, policies and practices and promoting appropriate legislation, policies and action in this regard	10.3.L Proportion of seats held by women in parliaments (national and provincial people's congress in China)	1	the National People's Congress of the People's Republic of China[13]	Sustainable Development Solutions Network, 2015[25]	2003-2017

	10.4 Adopt policies, especially fiscal, wage and social protection policies, and progressively achieve greater equality	10.4.1 Wages as a proportion of GDP	1	China Statistical Yearbook[2]	Xu et al., 2020[22]	2002-2009, 2011-2020
SDG11 Sustainable cities and Communities	11.1 By 2030, ensure access for all to adequate, safe and affordable housing and basic services and upgrade slums	11.1.L Percentage of urban population below minimum living guarantee	-1	China Statistical Yearbook[2]	Xu et al., 2020[22]	2000-2009, 2011-2020
	11.2 By 2030, provide access to safe, affordable, accessible and sustainable transport systems for all, improving road safety, notably by expanding public transport, with special attention to the needs of those in vulnerable situations, women, children, persons with disabilities and older persons	11.2.1 Passenger volumes (per capita)	1	China Statistical Yearbook[2]	Xu et al., 2020[22]	2002-2020
	11.5 By 2030, significantly reduce the number of deaths and the number of people affected and substantially decrease the direct economic losses relative to global gross domestic product caused by disasters, including water-related disasters, with a focus on protecting the poor and people in vulnerable situations	11.5.1 Number of deaths, missing persons and directly affected persons attributed to disasters per 100,000 population	-1	China Statistical Yearbook on Environment, China Statistical Yearbook[2,4]	United Nations Statistics Division, 2023[23]	2004-2020
		11.5.2 Direct economic loss in relation to global GDP, damage to critical infrastructure and number of disruptions	-1	China Statistical Yearbook on Environment, China Statistical Yearbook[2,4]	United Nations Statistics Division, 2023[23]	2004-2020

		to basic services, attributed to disasters (% GDP)				
		11.5.3L Road traffic deaths per 100,000 population	-1	China Statistical Yearbook, China Civil Affairs' Statistical Yearbook [2,22]	Sustainable Developme nt Solutions Network, 2015[25]	2000-2020
	11.6 By 2030, reduce the adverse per capita environmental impact of cities, including by paying special attention to air quality and municipal and other waste management	11.6.1 Ratio of industrial solid waste generated to the waste regularly collected and utilized	-1	China Statistical Yearbook[2]	Author constructed	2000-2020
	11.7 By 2030, provide universal access to safe, inclusive and accessible, green and public spaces, in particular for women and children, older persons and persons with disabilities	11.7.1 Green area in park per capita (square meters per person)	1	China Statistical Yearbook[2]	Zhang et al., 2022[21]	2001-2020
SDG12 Respon sible Consum	12.2 By 2030, achieve the sustainable management and efficient use of natural resources	12.2.2 Resource (e.g., water, energy) consumption per capita	-1	China Energy Statistical Yearbook, China Statistical Yearbook[2,15]	Xu et al., 2020[22]	2000-2020

ption and Production	12.4 By 2020, achieve the environmentally sound management of chemicals and all wastes throughout their life cycle, in accordance with agreed international frameworks, and significantly reduce their release to air, water and soil in order to minimize their adverse impacts on human health and the environment	12.4.2 Hazardous waste generated per capita	-1	China Statistical Yearbook on Environment, China Statistical Yearbook [2,4]	Xu et al., 2020[22]	2000-2020
	12.5 By 2030, substantially reduce waste generation through prevention, reduction, recycling and reuse	12.5.1 Percentage of industrial solid waste recycled (%)	1	China Statistical Yearbook on Environment, China Statistical Yearbook [2,4]	Xu et al., 2020[22]	2000-2020
	12.a Support developing countries to strengthen their scientific and technological capacity to move towards more sustainable patterns of consumption and production	12.a.1 Installed renewable energy-generating capacity in developing countries (in watts per capita)	1	China Energy Statistical Yearbook, China Statistical Yearbook[2,15]	Author constructed	2000-2019
	12.b Develop and implement tools to monitor sustainable development impacts for sustainable tourism that creates jobs and promotes local culture and products	12.b.L Tourism direct GDP as a proportion of total GDP	1	Yearbook of China Tourism Statistics, China Statistical Yearbook [2,16]	Author constructed	2000-2020
SDG13 Climate Action	13.1 Strengthen resilience and adaptive capacity to climate-related hazards and natural disasters in all countries	13.1.1 Number of deaths, missing persons and directly affected persons attributed to	-1	China Statistical Yearbook on Environment, China Statistical	United Nations Statistics Division,	2004-2020

		disasters per 100,000 population		Yearbook[2,4]	2023[23]	
		13.1.3 Proportion of local governments that adopt and implement local disaster risk reduction strategies in line with national disaster risk reduction strategies	1	China Statistical Yearbook on Environment[5]	United Nations Statistics Division, 2023[23]	2001-2020
	13.2 Integrate climate change measures into national policies, strategies and planning	13.2.2 Total CO2 emissions per year (tCO2)	-1	China Statistical Yearbook, Shan. et al., 2018, 2020, Guan et al. 2021[2,19-21]	United Nations Statistics Division, 2023[23]	2000-2019
SDG14 Life below Water	14.1 By 2025, prevent and significantly reduce marine pollution of all kinds, in particular from land-based activities, including marine debris and nutrient pollution	14.1.L Ratio of wastewater discharged at ocean to marine areas with established rights	-1	China Marine Statistical Yearbook[23]	Xu et al., 2020[22]	2000-2004, 2006-2015
	14.5 By 2020, conserve at least 10 per cent of coastal and marine areas, consistent with national and international law and based on the best available scientific information	14.5.1 Ratio of marine protected area to marine area with established rights	1	China Marine Statistical Yearbook[23]	Xu et al., 2020[22]	2000-2001, 2003-2004, 2006-2018

	14.a Increase scientific knowledge, develop research capacity and transfer marine technology, taking into account the Intergovernmental Oceanographic Commission Criteria and Guidelines on the Transfer of Marine Technology, in order to improve ocean health and to enhance the contribution of marine biodiversity to the development of developing countries, in particular small island developing States and least developed countries	14.a.1 Personnel in R&D in the field of ocean study (per million inhabitants)	1	China Marine Statistical Yearbook [23]	Xu et al., 2020[22]	2000-2018
SDG15 Life on Land	15.1 By 2020, ensure the conservation, restoration and sustainable use of terrestrial and inland freshwater ecosystems and their services, in particular forests, wetlands, mountains and drylands, in line with obligations under international agreements	15.1.1 Forest area as a proportion of total land area	1	China Forestry Statistical Yearbook, [24]	United Nations Statistics Division, 2023[23]	2002-2020
		15.1.2 Wetland area as a proportion of total land area	1	China Statistical Yearbook on Environment, China Statistical Yearbook [2,4]	Xu et al., 2020[22]	2001-2019
	15.2 By 2020, promote the implementation of sustainable management of all types of forests, halt deforestation, restore degraded forests and substantially increase afforestation and reforestation	15.2.1 Afforestation area as a percent of forest area	1	China Forestry Statistical Yearbook, [24]	Xu et al., 2020[22]	2002-2020

	globally					
	15.3 By 2030, combat desertification, restore degraded land and soil, including land affected by desertification, drought and floods, and strive to achieve a land degradation-neutral world	15.3.1 Proportion of land that is degraded over total land area	-1	China Statistical Yearbook on Environment[4]	United Nations Statistics Division, 2023[23]	2004-2020
	15.a Mobilize and significantly increase financial resources from all sources to conserve and sustainably use biodiversity and ecosystems	15.a.1 Official development assistance and public expenditure on conservation and sustainable use of biodiversity and ecosystems as a proportion of GDP	1	China Forestry Statistical Yearbook, China Statistical Yearbook[2,24]	United Nations Statistics Division, 2023[23]	2002-2018
	15.b Mobilize significant resources from all sources and at all levels to finance sustainable forest management and provide adequate incentives to developing countries to advance such management, including for conservation and reforestation	15.b.1 Government expenditure on environmental pollution control as a proportion of GDP	1	China Statistical Yearbook on Environment, China Statistical Yearbook[2,4]	Xu et al., 2020[22]	2004-2017
SDG16 Peace, Justice and	16.6 Develop effective, accountable and transparent institutions at all levels	16.6.1 Primary government expenditures as a proportion of original	1	Finance Yearbook of China[11]	United Nations Statistics Division,	2000-2020

Strong Institutions		approved budget			2023[23]	
	16.7 Ensure responsive, inclusive, participatory and representative decision-making at all levels	16.7.1 Proportions of positions (population groups) in public institutions (national and local legislatures, public service, and judiciary) compared to national distributions	1	National People's Congress of the People's Republic of China[13]	United Nations Statistics Division, 2023[23]	2003-2017
	16.9 By 2030, provide legal identity for all, including birth registration	16.9.L Proportion of children under 3 years of age whose births have been registered with a civil authority	1	China Health Statistical Yearbook [6]	Author constructed	2002-2020
	16.b Promote and enforce non-discriminatory laws and policies for sustainable development	16.b.L Proportion of seats held by women in parliaments (national and provincial people's congress in China)	1	the National People's Congress of the People's Republic of China[13]	Author constructed	2003-2017
SDG17 Partnerships for the Goals	17.1 Strengthen domestic resource mobilization, including through international support to developing countries, to improve domestic capacity for tax and other revenue collection	17.1.1 Total government revenue as a proportion of GDP	1	China Statistical Yearbook, Finance Yearbook of China [2,11]	United Nations Statistics Division, 2023[23]	2000-2020
		17.1.2 The percentage share of tax revenues in a country's gross domestic product (GDP)	1	Finance Yearbook of China, China Statistical Yearbook [2,11]	Author constructed	2003-2020
	17.3 Mobilize additional financial resources for developing countries from	17.3.1 Foreign direct investments (FDI) proportion of total	1	Finance Yearbook of China, China Statistical Yearbook	United Nations Statistics	2000-2020

	multiple sources	domestic budget		[2,11]	Division, 2023[23]	
		17.3.2 Foreign direct investments (FDI) as a proportion of total GDP	1	Finance Yearbook of China, China Statistical Yearbook [2,11]	Xu et al., 2020[22]	2000-2020
	17.8 Fully operationalize the technology bank and science, technology and innovation capacity-building mechanism for least developed countries by 2017 and enhance the use of enabling technology, in particular information and communications technology	17.8.1 Proportion of individuals using the Internet	1	China Statistical Yearbook [2]	United Nations Statistics Division, 2023[23]	2011-2020
	17.14 Enhance policy coherence for sustainable development	17.14.L Government health and education spending (% GDP)	1	Finance Yearbook of China, China Statistical Yearbook [2,11]	Sustainable Developme nt Report 2022[24]	2000-2020
	17.15 Respect each country's policy space and leadership to establish and implement policies for poverty eradication and sustainable development	17.15.L Expenditure on social security and employment (% total government expenditure)	1	Finance Yearbook of China, China Statistical Yearbook [2,11]	Xu et al., 2020[22]	2000-2020

Table 3.

The regional division in China following Resource and Environment Science and Data Center, Chinese Academy of Science[25]

Regions	Provinces
Southwest	Tibet, Sichuan, Chongqing, Guizhou, Yunnan
East	Shanghai, Jiangsu, Zhejiang, Anhui, Jiangxi, Shandong, Fujian
Central South	Henan, Hubei, Hunan, Guangxi, Guangdong, Hainan
North	Inner Mongolia, Beijing, Tianjin, Hebei, Shanxi
Northwest	Xinjiang, Gansu, Qinghai, Ningxia, Shaanxi
Northeast	Heilongjiang, Jilin, Liaoning

Supplementary text

Supplementary results on the priorities of SDGs at the national level

We also found that China achieved great co-benefits on most of the SDGs at the national level, such as SDG1 (No Poverty), SDG2 (Zero Hunger), SDG3 (Good Health and Well-being), SDG4 (Quality Education), SDG6 (Clean Water and Sanitation), SDG7 (Affordable and Clean Energy), SDG8 (Decent Work and Economic Growth), SDG9 (Industry, Innovation and Infrastructure), SDG10 (Reduced Inequalities), SDG11 (Sustainable cities and Communities), SDG14 (Life below Water) and SDG15 (Life on Land).

Supplementary results on the priority of SDGs at the provincial level

Among the 19 provinces, 14 of them had the highest trade-off in SDG5 (Gender Equality) (Anhui, Fujian, Gansu, Guangxi, Guizhou, Hainan, Hebei, Hunan, Jiangsu, Liaoning, Shandong, Sichuan, Yunnan, and Zhejiang) and 5 of them had the highest trade-off in SDG13 (Climate Action) (Inner Mongolia, Jiangxi, Ningxia, Shaanxi, Shanxi).

Among the 24 provinces, 14 of them had the highest synergy in SDG1 (No Poverty) (Anhui, Beijing, Fujian, Gansu, Guangdong, Guangxi, Hainan, Hebei, Hunan, Liaoning, Qinghai, Shaanxi, Shanxi, Yunnan) and 10 of them had the highest synergy in SDG6 (Clean Water and Sanitation) (Chongqing, Guizhou, Heilongjiang, Hubei, Jiangsu, Ningxia, Shandong, Shanghai, Tianjin, Zhejiang).

Reference

1. China Statistics Press, “Poverty Monitoring Report of Rural China” (Department of Household Surveys of the National Bureau of Statistics of China, 2020).
2. China Statistics Press, “China Statistical Yearbook” (National Bureau of Statistics of the People's Republic of China, 2001-2021) [in Chinese].
3. Press of Yearbook of China's Insurance, “Yearbook of China's Insurance” (China Insurance Regulatory Commission, 2001-2021).
4. China Statistics Press, “China Statistical Yearbook on Environment” (National Bureau of Statistics & State Environmental Protection Administration of the People's Republic of China, 2001-2021) [in Chinese].
5. Friends of Nature. China Environmental Yearbook (in Chinese). (Brill, 2001-2021).
6. Pecking Union Medical College Press, “China Health Statistical Yearbook” (Ministry of Health of the People's Republic of China, 2001-2021) [in Chinese].
7. China Statistics Press, “China Population & Employment Statistic Yearbook” (Department of Population and Employment Statistics of the National Bureau of Statistics of China, 2007-2021) [in Chinese].
8. China Statistics Press, “China Rural Statistical Yearbook” (National Bureau of Statistics of the People's Republic of China, 2001-2021) [in Chinese].
9. China Statistics Press, “China Statistical Yearbook on Science and Technology” (National Bureau of Statistics of the People's Republic of China & Ministry of Science and Technology of China, 2001-2021) [in Chinese].
10. China Statistics Press, “China Health and Family Planning Statistical Yearbook” (National Bureau of Statistics of the People's Republic of China, 2001-2021) [in Chinese].
11. China Financial & Economic Publishing House, “Finance Yearbook of China” (Ministry of Finance of the People's Republic of China, 2001-2021) [in Chinese].
12. People's Education Press, “Educational Statistics Yearbook of China” (Ministry of Education of the People's Republic of China, 2001-2021) [in Chinese].
13. The National People's Congress of the People's Republic of China, <http://www.npc.gov.cn> (2022).
14. China Statistics Press, “China Urban Construction Statistical Yearbook” (Ministry of Housing Urban-Rural Development People’s Republic of China, 2003-2021) [in Chinese].

15. China Statistics Press, “China Energy Statistical Yearbook” (National Bureau of Statistics of the People's Republic of China, 2001-2021) [in Chinese].
16. China Tourism Press, “Yearbook of China Tourism Statistics” (National Tourism Administration of the People's Republic of China, 2001-2021) [in Chinese].
17. China Communications Press, “Yearbook of China Transportation & Communications” (The National Development and Reform Commission of the People's Republic of China, 2001-2021) [in Chinese].
18. China Statistics Press, “China Industry Statistical Yearbook” (National Bureau of Statistics of the People's Republic of China, 2001-2021) [in Chinese].
19. Shan, Y. et al. China CO 2 emission accounts 1997–2015. *Sci. Data* **5**, 170201 (2018).
20. Shan, Y. et al. China CO 2 emission accounts 2016-2017. *Sci. Data* **5**, 170201 (2020).
21. Zhang J, Wang S, Pradhan P, et al. Untangling the interactions among the Sustainable Development Goals in China. *Sci. Bull.* **67(9)**, 977-984 (2022).
22. Xu Z., et al. Assessing progress towards sustainable development over space and time. *Nature* **577**, 74–78, https://doi.org/10.1038/s41586-019-1846-3 (2020).
23. Inter-agency and Expert Group on SDG Indicators, (IAEG-SDGs), Tier Classification for Global SDG Indicators, https://unstats.un.org/sdgs/files/Tier%20Classification%20of%20SDG%20Indicators_31%20Mar%202023_web.pdf (2023).
24. Sachs, J., Lafortune, G., Kroll, C., Fuller, G., Woelm, F.. From Crisis to Sustainable Development: the SDGs as Roadmap to 2030 and Beyond. Sustainable Development Report 2022, Cambridge: Cambridge University Press, doi.org/10.1017/9781009210058 (2022).
25. Sustainable Development Solutions Network, Indicators and a Monitoring Framework for the Sustainable Development Goals: Launching a Data Revolution for the SDGs (2015).
