# Supplementary material for: Intranational synergies and trade-offs reveal common and differentiated priorities of sustainable development goals in China
Source: Nat Commun. 2024 Mar 13;15:2251. doi: 10.1038/s41467-024-46491-6 (PMC10937989; doi:10.1038/s41467-024-46491-6)
Supplement: Supplementary file 3 — Description of Additional Supplementary Files [file 41467_2024_46491_MOESM3_ESM.pdf]

Description of Additional Supplementary Files

File Name: Supplementary Data 1
Description: -Source DataSupplementary Information_R2

File Name: Supplementary Data 2
Description: Explanations on the association between indicators_R2

File Name: Supplementary Data 3
Description: Source Data-Main text_R2

File Name: Supplementary Data 4
Description: Synergies and tradeoffs _R2

File Name: Supplementary Data 5
Description: Original Data_R2
