# Supplementary material for: Intranational synergies and trade-offs reveal common and differentiated priorities of sustainable development goals in China
Source: Nat Commun. 2024 Mar 13;15:2251. doi: 10.1038/s41467-024-46491-6 (PMC10937989; doi:10.1038/s41467-024-46491-6)
Supplement: Supplementary file 9 — Reporting Summary [file 41467_2024_46491_MOESM9_ESM.pdf]

nature portfolio

Corresponding author(s): Chaoyang Wu, Fang Chen

Last updated by author(s): Feb 13, 2024

Reporting Summary

Nature Portfolio wishes to improve the reproducibility of the work that we publish. This form provides structure for consistency and transparency in reporting. For further information on Nature Portfolio policies, see our Editorial Policies and the Editorial Policy Checklist.

Statistics

For all statistical analyses, confirm that the following items are present in the figure legend, table legend, main text, or Methods section.

n/a	Confirmed	
☐	☒	The exact sample size (*n*) for each experimental group/condition, given as a discrete number and unit of measurement
☒	☐	A statement on whether measurements were taken from distinct samples or whether the same sample was measured repeatedly
☐	☒	The statistical test(s) used AND whether they are one- or two-sided<br>*Only common tests should be described solely by name; describe more complex techniques in the Methods section.*
☒	☐	A description of all covariates tested
☐	☒	A description of any assumptions or corrections, such as tests of normality and adjustment for multiple comparisons
☐	☒	A full description of the statistical parameters including central tendency (e.g. means) or other basic estimates (e.g. regression coefficient) AND variation (e.g. standard deviation) or associated estimates of uncertainty (e.g. confidence intervals)
☐	☒	For null hypothesis testing, the test statistic (e.g. F, t, r) with confidence intervals, effect sizes, degrees of freedom and P value noted<br>*Give P values as exact values whenever suitable.*
☒	☐	For Bayesian analysis, information on the choice of priors and Markov chain Monte Carlo settings
☒	☐	For hierarchical and complex designs, identification of the appropriate level for tests and full reporting of outcomes
☐	☒	Estimates of effect sizes (e.g. Cohen's d, Pearson's r), indicating how they were calculated

Our web collection on statistics for biologists contains articles on many of the points above.

Software and code

Policy information about availability of computer code

Data collection	No code or software was used in data collection.
Data analysis	All R scripts and excel files used to process the data are available from the corresponding authors upon reasonable request.

For manuscripts utilizing custom algorithms or software that are central to the research but not yet described in published literature, software must be made available to editors and reviewers. We strongly encourage code deposition in a community repository (e.g. GitHub). See the Nature Portfolio guidelines for submitting code & software for further information.

Data

Policy information about availability of data

All manuscripts must include a data availability statement. This statement should provide the following information, where applicable:

- Accession codes, unique identifiers, or web links for publicly available datasets
- A description of any restrictions on data availability
- For clinical datasets or third party data, please ensure that the statement adheres to our policy

The authors declare that the data supporting the findings of this study are available within the paper and its supplementary files. Source data are provided with this paper.

Research involving human participants, their data, or biological material

Policy information about studies with human participants or human data. See also policy information about sex, gender (identity/presentation), and sexual orientation and race, ethnicity and racism.

Reporting on sex and gender	n.a.
Reporting on race, ethnicity, or other socially relevant groupings	n.a.
Population characteristics	n.a.
Recruitment	n.a.
Ethics oversight	n.a.

Note that full information on the approval of the study protocol must also be provided in the manuscript.

Field-specific reporting

Please select the one below that is the best fit for your research. If you are not sure, read the appropriate sections before making your selection.

☐ Life sciences ☐ Behavioural & social sciences ☒ Ecological, evolutionary & environmental sciences

For a reference copy of the document with all sections, see nature.com/documents/nr-reporting-summary-flat.pdf

Ecological, evolutionary & environmental sciences study design

All studies must disclose on these points even when the disclosure is negative.

Study description	We reveal the common but differentiated priorities of the 17 sustainable development goals (SDGs) for China at provincial, regional and national levels through interaction networks, using a unified annual dataset of 102 indicators (118 original) at the national and subnational levels simultaneously.
Research sample	We built built synergy and trade-off networks at the national and sub-national levels, respectively. The synergy and trade-off intensity was set to be the weighted edge and the hub score of the 17 goals were in the nodes in the networks. We analyzed the hub score to determine which goal served as the central hub in the synergy and trade-off networks. The larger the hub score, the more important the node as the central hub in the networks was. We analyzed these variations at national, provincial and regional levels among the 17 goals.
Sampling strategy	n.a. (no sampling was conducted. The dataset used in this study was collected directly from the official database for the 31 provinces and the whole country)
Data collection	Data for the selected indicators in this study were obtained from the following official sources: the National Bureau of Statistics of the People's Republic of China, the China Statistical Yearbook, the Finance Yearbook of China, the China Statistical Yearbook on the Environment, the Educational Statistics Yearbook of China, the China Health Statistics Yearbook, the China Energy Statistical Yearbook, and other 9 Yearbooks from various ministries, such as insurance, urban construction, tourism, transportation & communications, industry, civil affairs, marine, forestry and population.
Timing and spatial scale	The data overall covers the time period from 2000 to 2020 with varied coverage at the national and subnational levels.
Data exclusions	No data were excluded in this study.
Reproducibility	All attempts to repeat the results were successful.
Randomization	n.a. (This study does not involve group allocation and sample randomization)
Blinding	n.a.(This study does not involve group allocation and investigator blinding)

Did the study involve field work? ☐ Yes ☒ No

Reporting for specific materials, systems and methods

We require information from authors about some types of materials, experimental systems and methods used in many studies. Here, indicate whether each material, system or method listed is relevant to your study. If you are not sure if a list item applies to your research, read the appropriate section before selecting a response.

Materials & experimental systems

n/a	Involved in the study
☒	☐ Antibodies
☒	☐ Eukaryotic cell lines
☒	☐ Palaeontology and archaeology
☒	☐ Animals and other organisms
☒	☐ Clinical data
☒	☐ Dual use research of concern
☒	☐ Plants

Methods

n/a	Involved in the study
☒	☐ ChIP-seq
☒	☐ Flow cytometry
☒	☐ MRI-based neuroimaging

Plants

Seed stocks	n.a.
Novel plant genotypes	n.a.
Authentication	n.a.
